# Prediction of Lymph-Node Metastasis in Cancers Using Differentially Expressed mRNA and Non-coding RNA Signatures

**DOI:** 10.3389/fcell.2021.605977

**Published:** 2021-02-11

**Authors:** Shihua Zhang, Cheng Zhang, Jinke Du, Rui Zhang, Shixiong Yang, Bo Li, Pingping Wang, Wensheng Deng

**Affiliations:** ^1^College of Life Science and Health, Wuhan University of Science and Technology, Wuhan, China; ^2^State Key Laboratory of Tea Plant Biology and Utilization, Anhui Agricultural University, Hefei, China; ^3^Central Laboratory, Xiaogan Hospital Affiliated to Wuhan University of Science and Technology, Xiaogan, China; ^4^School of Computer Science and Technology, Wuhan University of Science and Technology, Wuhan, China; ^5^School of Life Science and Technology, Harbin Institute of Technology, Harbin, China

**Keywords:** lymph-node metastasis, molecular profiles, classifiers, webserver, biomarker

## Abstract

Accurate prediction of lymph-node metastasis in cancers is pivotal for the next targeted clinical interventions that allow favorable prognosis for patients. Different molecular profiles (mRNA and non-coding RNAs) have been widely used to establish classifiers for cancer prediction (e.g., tumor origin, cancerous or non-cancerous state, cancer subtype). However, few studies focus on lymphatic metastasis evaluation using these profiles, and the performance of classifiers based on different profiles has also not been compared. Here, differentially expressed mRNAs, miRNAs, and lncRNAs between lymph-node metastatic and non-metastatic groups were identified as molecular signatures to construct classifiers for lymphatic metastasis prediction in different cancers. With this similar feature selection strategy, support vector machine (SVM) classifiers based on different profiles were systematically compared in their prediction performance. For representative cancers (a total of nine types), these classifiers achieved comparative overall accuracies of 81.00% (67.96–92.19%), 81.97% (70.83–95.24%), and 80.78% (69.61–90.00%) on independent mRNA, miRNA, and lncRNA datasets, with a small set of biomarkers (6, 12, and 4 on average). Therefore, our proposed feature selection strategies are economical and efficient to identify biomarkers that aid in developing competitive classifiers for predicting lymph-node metastasis in cancers. A user-friendly webserver was also deployed to help researchers in metastasis risk determination by submitting their expression profiles of different origins.

## Introduction

Regional lymph-node metastasis is an important predictor for tumor recurrence and survival in patients with aggressive cancers (Hermanek, 2000; Xu et al., 2018). The diagnosis of lymphatic metastasis in a certain cancer may be uncertain even after extensive clinical examinations, such as endosonography, magnetic resonance imaging, and computed tomography (Christensen et al., 2006; Obinu et al., 2018; Zeng et al., 2019). Cancer patients examined with ambiguous lymphatic metastasis usually suffer from uncontrolled disease progression and a short overall survival period (Biaoxue et al., 2011; Yang et al., 2019a). Though the prognosis depends on different factors including tumor cell type, primary site, dissemination ability, clinical intervention, and drug response, the low survival rate of patients may be mainly attributed to the unclear determination of lymph-node metastasis (Li et al., 2019; Sugimura and Yoshimura, 2019). Therefore, it is of great importance to accurately predict regional lymphatic metastasis in cancerous patients for early tumor spread detection and appropriate clinical decision-making.

With rapid development of high-throughput molecular profiling technologies, large amounts of expression data [mRNA and non-coding RNA (ncRNA) that includes microRNA (miRNA) and long non-coding RNA (lncRNA)] have been generated and are publicly available, which facilitate forecasting paradigms in tumor origin, cancerous or non-cancerous state, and cancer subtype by using these profiles (Perez-Diez et al., 2007; Rosenfeld et al., 2008; Jiang et al., 2009, 2010; Monzon et al., 2010; Varadhachary, 2013; Flippot et al., 2016). As a key prognostic factor in cancer prediction, lymphatic metastasis has also been evaluated in several attempts based on these molecular profiles and now become useful diagnostic algorithms (Moriya et al., 2009; Qu et al., 2018; Ma et al., 2019).

As well-recognized genetic biomarkers in cancers, genes have been large-scale profiled at the transcriptional level (mRNA) and the expression profiling has been used in several studies for lymph-node metastasis evaluation (Kikuchi et al., 2003; Wang et al., 2005). For example, Zhou et al. proposed a mRNA-based logistic regression model to discriminate lymph-node metastatic and non-metastatic cases in patients with oral tongue squamous cell carcinoma (Zhou et al., 2006). This classifier showed a high overall accuracy rate of 85% with a small number of gene markers. The expression profiles of miRNAs that are small non-coding RNAs regulating the expression of genes involved in biological processes such as tumor cell proliferation, migration, and invasion have also been utilized to predict lymph-node metastasis in cancers (Zhang et al., 2016; Cheng et al., 2018). For example, a recent study reported a miRNA classifier that screened a 4-miRNA signature based on differential expression analysis and quantitative expression validation and achieved a perfect sensitivity and specificity in lymph-node metastasis evaluation for breast cancer patients (Chen et al., 2018). LncRNAs are newly identified long non-coding RNAs that act as complicated regulatory roles in diverse biological processes (Alvarez-Dominguez et al., 2012; Ulitsky and Bartel, 2013; Fatica and Bozzoni, 2014; Jiang et al., 2014, 2015; Liu et al., 2018; Cheng et al., 2019) and even cancers (Gutschner and Diederichs, 2012; Mitobe et al., 2018; Wang et al., 2019). Sørensen *et al*. demonstrated the potentiality of forecasting lymphatic metastasis in breast cancer using lncRNA profiles (Sorensen et al., 2015). The authors established a lncRNA classifier based on support vector machine (SVM) algorithm that gained a high overall accuracy in prediction of lymphatic metastasis in breast cancer patients.

To our knowledge, the existing studies mostly applied a retrospective in-hospital strategy that seems to be procedure-tedious in patient surveying. In this process, several limiting factors such as individual difference, environmental change, and differentiated clinical management may be origins of noise and ultimately affect the classification performance (Bur et al., 2019; Reijnen et al., 2019). Instead, large samples of different molecular profiles available in public serve as useful resources for the development of machine leaning methods in lymph-node metastasis evaluation by computational biologists. As seen in existing studies, only a few types of common cancers have been focused. Indeed, large-scale genome sequencing projects (e.g., The Cancer Genome Atlas Program abbreviated as TCGA) for most cancers have been performed and thus all of them should be scheduled in clinical application. In addition, systematical comparison and evaluation of classifiers based on different profiles and algorithms may be necessary prior to establishment of promising prediction platforms.

With the above considerations, we established SVM classifiers based on different profiles to predict lymphatic metastasis in a spectrum of cancers. For these classifiers, novel feature selection strategies were adopted to screen differentially expressed signatures between lymph-node metastatic and non-metastatic groups in cancers. A total of 2,491 mRNA, 2,364 miRNA, and 2,491 lncRNA expression datasets were retrieved from TCGA to develop classifiers in nine representative cancers. The efficiency of these SVM classifiers was revealed having an overall accuracy of 81.25% on different profiles with small biomarker sets (seven biomarkers on average). We also compared these SVM classifiers with two other benchmark classifiers (K-Nearest Neighbor, KNN; Random Forest, RF) based on the same profiles, and our results showed that SVM classifiers had the better performance. To enable researchers to predict lymph-node metastasis in tumor samples of their interest, we made these SVM classifiers publicly available through an interface-concise webserver named LNMpredictor (http://lnmpredictor.wchoda.com).

## Materials and Methods

### Cancer Screening and Data Collection

**Figure 1:**
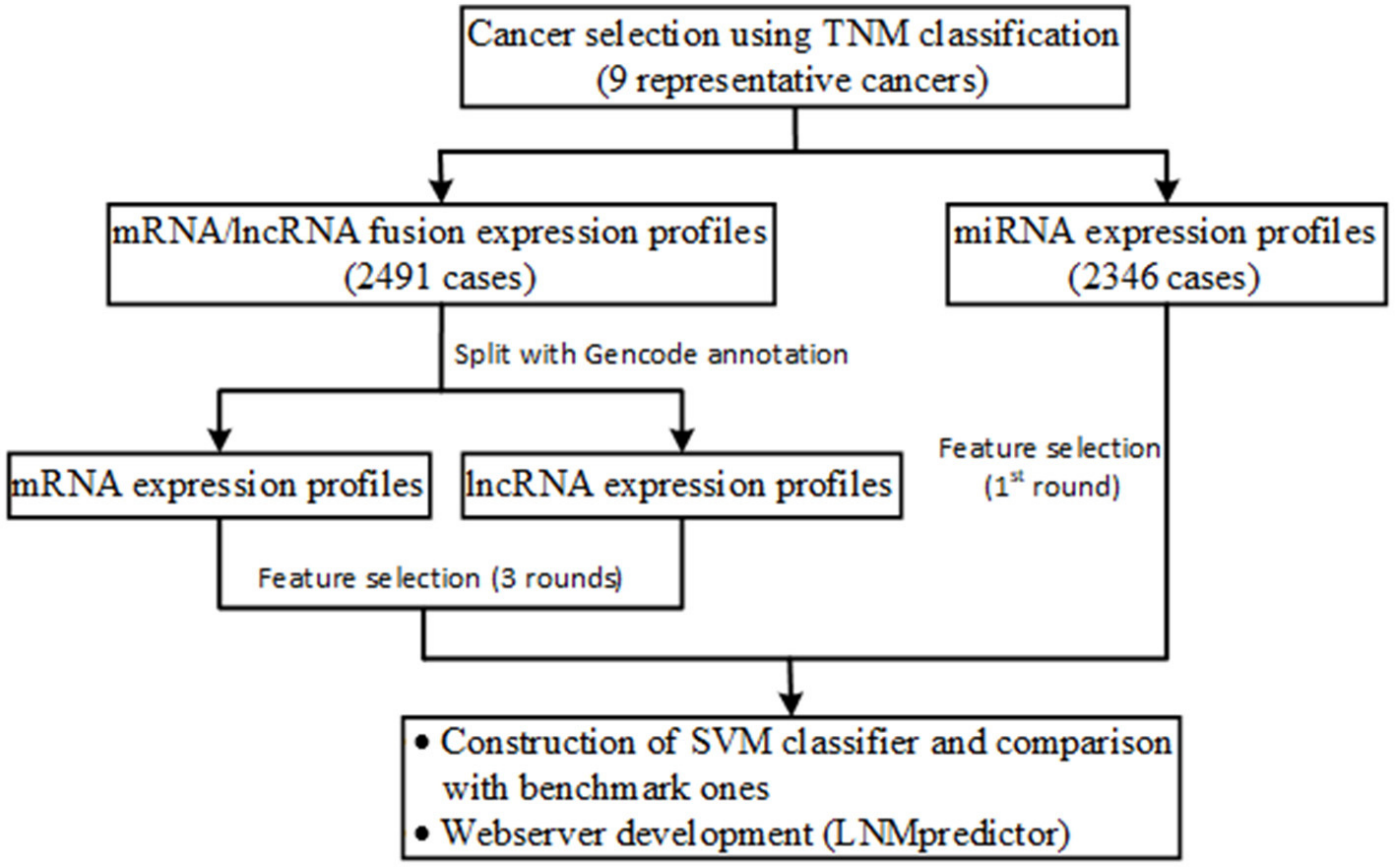
Schematic illustration of the workflow of data collection, analysis, classifier construction, and webserver development.

Figure 1 shows the flowchart of our data collection, analysis, classifier construction, and webserver development. We firstly used the clinical TNM (Tumor, Node, and Metastatic classification index) staging data from TCGA to screen those cancers that have definite lymph-node metastasis in patients. In detail, cancers with an N- and T-index of 1–4 and an M-index of 0 were determined as lymph-node metastatic cases, and cancers with an N- and M-index of 0 and a T-index of 1–4 were determined as non-metastatic controls. It is notable that the cases with an M-index of 1–4 were filtered out to avoid the possible noise in modeling of lymph-node metastasis evaluation because distant organ metastasis co-existed with regional lymphatic metastasis in these cases. In total, nine types of cancers with clear TNM-based lymphatic metastasis classification information were retained. For these selected cancers, 2,491 mRNA, 2,364 miRNA, and 2,491 lncRNA expression profiles including normal, lymph-node metastatic, and non-metastatic samples were collected (see details in Table 1; note that sufficient samples of more than 10 in the above three groups were required for subsequent feature selection). The samples sequenced for mRNA, miRNA, and lncRNA profiles were selected from the Illumina platform wherein miRNA expression was specifically sequenced with the BCGSC (IlluminaHiSeq_miRNAseq) sequencing platform (that facilitates highly sensitive and specific detection of common human miRNAs). All the clinical and expression data of patients were retrieved using customized functions implemented in the R package TCGAbiolinks (Colaprico et al., 2016) and handled with our in-house Python scripts.

**Table 1:** Sample number of each cancer for mRNA, miRNA, and lncRNA profile, feature selection, training, and testing datasets.

**Cancers**	**mRNA- or lncR-based datasets**					**miR-based datasets**				
	**NR**	**LNM**	**NM**	**Sets for feature selection**	**Sets for training/testing SVM classifiers**	**NR**	**LNM**	**NM**	**Sets for feature selection**	**Sets for training/testing SVM classifiers**
Bladder urothelial carcinoma	19	26	134	179	160	19	27	135	162	162
Breast invasive carcinoma	112	148	454	714	602	103	145	449	594	594
Cervical and endocervical cancers	3	27	80	110	107	3	27	80	107	107
Colon adenocarcinoma	41	41	242	324	283	8	38	224	262	262
Kidney renal clear cell carcinoma	72	11	201	284	212	71	12	198	210	210
Lung adenocarcinoma	59	53	231	343	284	46	53	221	274	274
Lung squamous cell carcinoma	49	40	259	348	299	45	39	242	281	281
Pancreatic adenocarcinoma	4	58	20	78	78	4	59	20	79	79
Rectum adenocarcinoma	10	18	79	107	97	3	18	75	93	93
Total	369	422	1,700	2,487	2,122	302	418	1,644	2,062	2,062

*miR, miRNA; lncR, lncRNA; NR, normal samples; LNM, lymph-node metastatic samples; NM, non-metastatic samples. mRNA- and lncR-based datasets had the same sample number*.

### Data Preprocessing

Gene expression quantification (a data type of TCGA) of mRNAs, miRNAs, and lncRNAs across samples was selected as molecular profiles for cancers as individual datasets. For each dataset of a given cancer, we discarded mRNAs, miRNAs, and lncRNAs that had missing values in more than 30% of all the samples. The remaining missing values were estimated using the impute.knn function implemented in the R package imput (http://www.bioconductor.org/packages/release/bioc/html/impute.html). Due to the fact that mRNAs and lncRNAs are fused as expression profiles for cancers in current TCGA sequencing platforms, we therefore separated them for their separate feature selection and profile-based classifier construction. For this purpose, in-house Python scripts were implemented based on the genomic annotation files (*gencode.v30.basic.annotation.gff3* and *gencode.v30.long_noncoding_RNAs.gtf* ) of human mRNAs and lncRNAs retrieved from Gencode (https://www.gencodegenes.org) that provides high-quality reference gene annotation with experimental validation for human genomes.

### Feature Selection

For each of the nine representative cancers, three rounds of feature extraction analysis were conducted for establishing practical classifiers that can achieve desirable classification performance with a small set of biomarkers (high relevance with target class and low redundancy in feature dimension), as follows: (1) we firstly screen differentially expressed mRNAs, miRNAs, and lncRNAs as biomarkers between lymph-node metastatic and non-metastatic groups in a certain cancer (significant *P* values were chosen the same as ≤ 0.01 for mRNA, miRNA, and lncRNA profiles); (2) from these biomarkers, differentially expressed ones between normal and diseased groups were re-screened, wherein the diseased group represented a pool of lymph-node metastatic and non-metastatic cases in a cancer (significant *P* values were set the same as in the first round of analysis); (3) finally, principal component analysis (PCA) was performed for dimensionality reduction if the re-screened biomarkers seemed high-dimensional. We used the R package DESeq2 that can estimate variance-mean dependence in count data from high-throughput sequencing assays and test for differential expression using the negative binomial distribution (Love et al., 2014), to identify differentially expressed mRNAs, miRNAs, or lncRNAs between case and control groups in the first two rounds of analysis, and dimensionality reduction was performed using the PCA function implemented in the Python package scikit-learn [a machine learning toolkit accessible at http://scikit-learn.org/stable/index.html (Swami and Jain, 2013)].

In the whole strategy, the first round of analysis ensures that the screened mRNA, miRNA, and lncRNA markers have strong relevance to lymph-node metastatic class in cancer samples, which is necessary for regional lymphatic metastasis evaluation. For this purpose, mRNAs, miRNAs, and lncRNAs with false discovery rate (FDR)-adjusted *P* ≤ 0.01 were screened as candidates that showed significantly different mean values between lymph-node metastatic and non-metastatic groups in certain cancers. We were also concerned about a prerequisite that the screened biomarkers should be related to a diseased state that involved lymph-node metastatic and non-metastatic substates in cancers. Therefore, the second round of analysis was implemented to discard ones that might have no roles in cancer progression and cause the possible bias in classification. From another perspective, this round of implementation can help lower the redundancy in feature dimension as that in the third round of analysis. Because of the much higher dimensionality of both mRNA and lncRNA profiles than that of miRNA profiles, we applied the PCA-based dimensionality reduction for mRNA and lncRNA profiles in our feature extraction analysis.

### Classifier Construction and Webserver Development

The screened mRNAs, miRNAs, and lncRNAs from the above feature selection were considered as differentially expressed biomarkers for cancer lymph-node metastasis prediction. In this study, SVM-based machine learning algorithm was adopted as the classifying model that has been demonstrated to have good performance in many classification cases with different types of molecular profiles (Hira and Gillies, 2015; Singh and Sivabalakrishnan, 2015; Huang et al., 2018; Liu et al., 2019). As described above, our proposed feature selection strategy guaranteed the acquisition of a small set of biomarkers with a high prediction performance that is a main objective of the research in cancer prediction, including cancer origin prediction, tumor subtype classification, and cancerous and non-cancerous sample determination. With the same biomarker set, SVM classifiers based on different profiles were systematically compared with other two commonly used benchmark classifiers, KNN and RF, for a more comprehensive evaluation of the SVM algorithm. As to the imbalanced samples of lymph-node metastatic and non-metastatic groups in cancers, we adopted an under-sampling strategy to achieve balanced datasets in these two groups, which can avoid the imbalance problem and improve the SVM classifier performance (Jiang et al., 2013; Hazan et al., 2018). After this, all individual models were trained with a fivefold cross-validation to improve their prediction performance. With the trained SVM models, we developed a Python-based webserver named LNMpredictor to enable users to predict lymph-node metastasis in cancers by uploading mRNA, miRNA, or lncRNA expression profiles of their own labs. The webserver was constructed using a freely available and open source framework, Django (https://www.djangoproject.com). The trained SVM classifying models were stored as individual files by joblib, a Python package named scikit-learn (https://scikit-learn.org). The corresponding web interface was deployed by uWSGI (https://uwsgi-docs.readthedocs.io) and Nginx (http://nginx.org).

## Results

### Cancer and Sample Statistics

In this study, we focused on those cancers with clear measurement of lymph-node metastasis and adequate samples size, to construct different profiles based on SVM classifiers. The majority of the nine selected cancers are adenocarcinomas (~70%) together with squamous cell and urothelial carcinomas (account for ~20%), covering a wide range of organs or tissues, such as breast, lung, kidney, colon, bladder, cervix uteri, pancreas, and rectum (a total of eight organ or tissue types). Therefore, our strategy ensured a complete representation of main cancer types defined by their anatomic tissues or original organs. Among the cancers, lung-derived adenocarcinomas and squamous cell carcinoma were both presented due to their high lymph-node metastasis risk in clinical cases (Zhong et al., 2018; Deng et al., 2019). For the following classifier establishment, a total of 2,491 mRNA samples, 2,364 miRNA samples, and 2,491 lncRNA samples were respectively selected, wherein normal, lymph-node metastatic, and non-metastatic cases were separated for each cancer type and cancer-specific profiles (Table 1).

### Feature Overview

**Figure 2:**
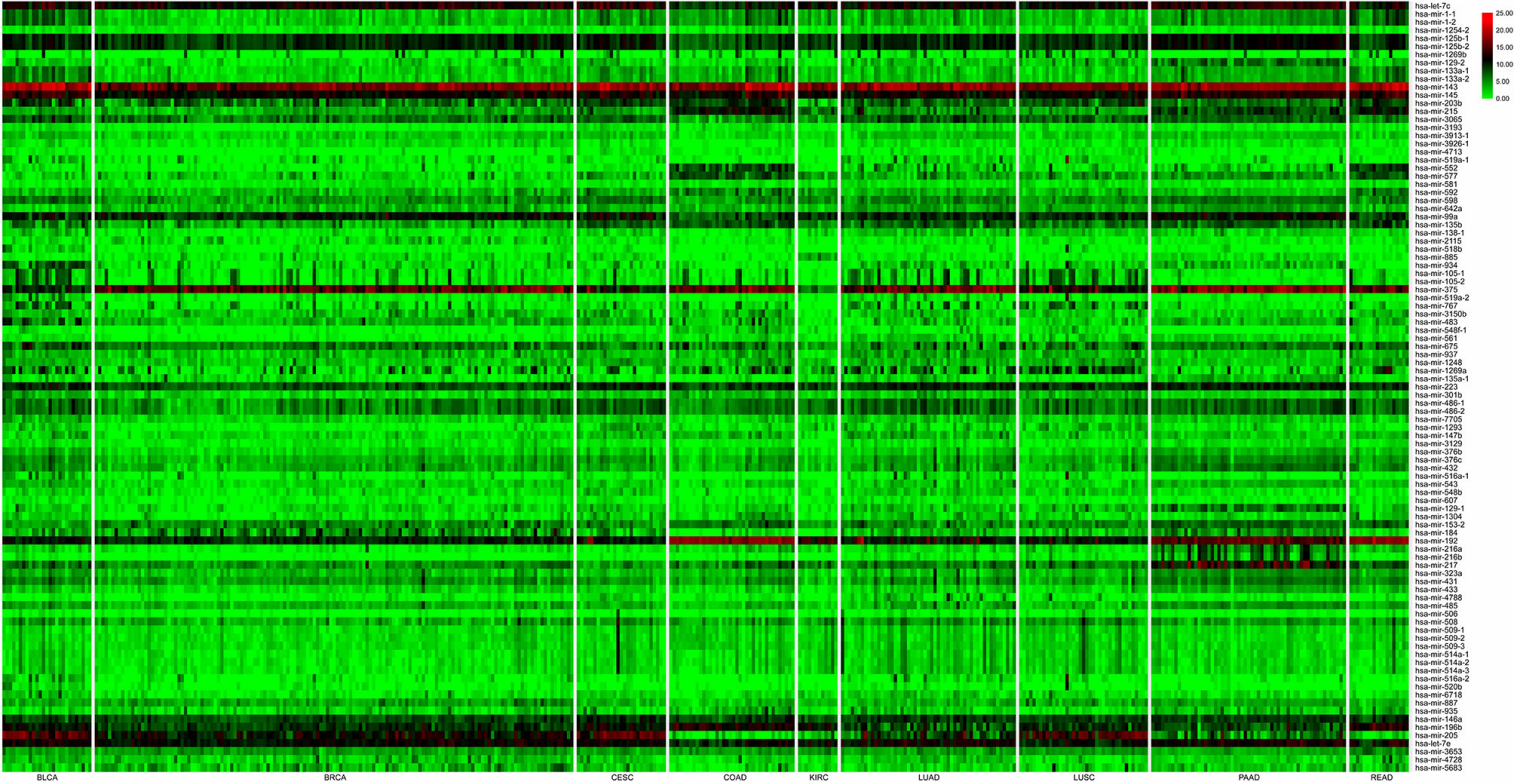
Expression heatmap of differentially expressed miRNAs for all the cancer samples (nine cancer types). In the plot, nine cancer types (rows) were indicated, namely, bladder urothelial carcinoma (BLCA), breast invasive carcinoma (BRCA), cervical and endocervical cancers (CESC), colon adenocarcinoma (COAD), kidney renal clear cell carcinoma (KIRC), lung adenocarcinoma (LUAD), lung squamous cell carcinoma (LUSC), pancreatic adenocarcinoma (PAAD), and rectum adenocarcinoma (READ), and differentially expressed miRNA signatures (columns) were top-down placed according to the left–right ordinal cancer types.

Identifying efficient features for lymph-node metastasis prediction in different cancers is a key step for the construction of classifiers with high performance. To achieve this goal, we used differentially expressed mRNAs and ncRNAs (miRNAs and lncRNAs) between lymph-node metastatic and non-metastatic groups in a cancer as biomarkers that can differentiate between patients with and without lymph-node metastasis. Another concern about the selected feature is their sizes in different cancers with a given molecular profile. Logically, the first two rounds of analysis is necessary for all the profile types in true biomarker extraction. Indeed, miRNA profile has a quite low dimension (~1,880) compared with that of mRNA (45,312-dimension) and lncRNA (15,171-dimension) profiles. Therefore, for miRNA-based datasets with 1,881 common miRNAs in different cancers, we only adopted the first round of analysis for feature selection that can screen differentially expressed miRNAs as biomarker set with appropriate size (3–27 features, with an average of 12 miRNAs as biomarkers in all cancers; see details in Table 2). For both mRNA- and lncRNA-based datasets, the first two rounds of analysis were initially conducted. We observed that comparative numbers of biomarkers were obtained in the two rounds of analysis (see details in Table 2). However, the size of biomarker sets extracted from mRNA and lncRNA profiles in different cancers seemed unpractical with an average of 591 and 276 features. Therefore, the third round of analysis (PCA-based feature reduction) was implemented. After this reduction, we saw a similar size of biomarkers for mRNA and lncRNA profiles (six and four biomarkers on average), which is smaller than that in miRNA profile. Detailed information regarding the final extracted mRNA, miRNA, and lncRNA features is available in Supplementary Table 1. Among the screened mRNA and ncRNA features, miRNA biomarkers represented the real molecular entities because PCA-based feature reduction was not conducted in this study. We constructed an expression heatmap of differentially expressed miRNAs for all the cancer samples to demonstrate the rationality of our feature selection method, and the resulting Figure 2 showed the clear distinction of some cancer types with others due to their differentially expressed miRNA signatures in diverse cancer types.

**Table 2:** Number of mRNA, miR, and lncR signatures in feature selection and SVM performance using mRNA, miR, and lncR profiles.

**Cancers**	**No. of mRNA signatures**			**mRNA-based SVM classifiers**		**No. of miR signatures (first round)**	**miR-based SVM classifiers**		**No. of lncR signatures**			**lncR-based SVM classifiers**	
	**First round**	**Second round**	**Third round**	**Training_ Acc (%)**	**Testing_ Acc (%)**		**Training_ Acc (%)**	**Testing_ Acc (%)**	**First round**	**Second round**	**Third round**	**Training_ Acc (%)**	**Testing_ Acc (%)**
Bladder urothelial carcinoma	1,176	500	8	89.29	77.08	27	100	77.55	496	165	7	88.39	81.25
Breast invasive carcinoma	1,629	834	6	79.81	67.96	8	76.39	73.74	875	410	12	79.33	69.61
Cervical and endocervical cancers	220	40	3	70.27	84.85	6	71.62	81.82	45	6	3	70.27	84.85
Colon adenocarcinoma	706	559	3	84.85	87.06	6	90.16	88.61	459	373	3	86.87	87.06
Kidney renal clear cell carcinoma	660	414	6	98.65	92.19	8	95.24	95.24	215	123	2	97.30	84.38
Lung adenocarcinoma	3,495	2,465	6	80.81	84.88	14	88.48	79.52	2,146	1,272	2	79.80	84.88
Lung squamous cell carcinoma	443	304	18	87.08	90.00	29	93.88	84.71	167	113	2	86.12	90.00
Pancreatic adenocarcinoma	56	×	2	75.93	75.00	3	80.00	70.83	14	×	2	75.93	75.00
Rectum adenocarcinoma	293	200	3	88.06	70.00	11	92.31	85.71	51	21	2	86.57	70.00
Overall accuracy				83.86	81.00		87.56	81.97				83.40	80.78

*miR, miRNA; lncR, lncRNA; NR, normal samples; LNM, lymph-node metastatic samples; NM, non-metastatic samples*.

### Classifiers Performance Evaluation

**Figure 3:**
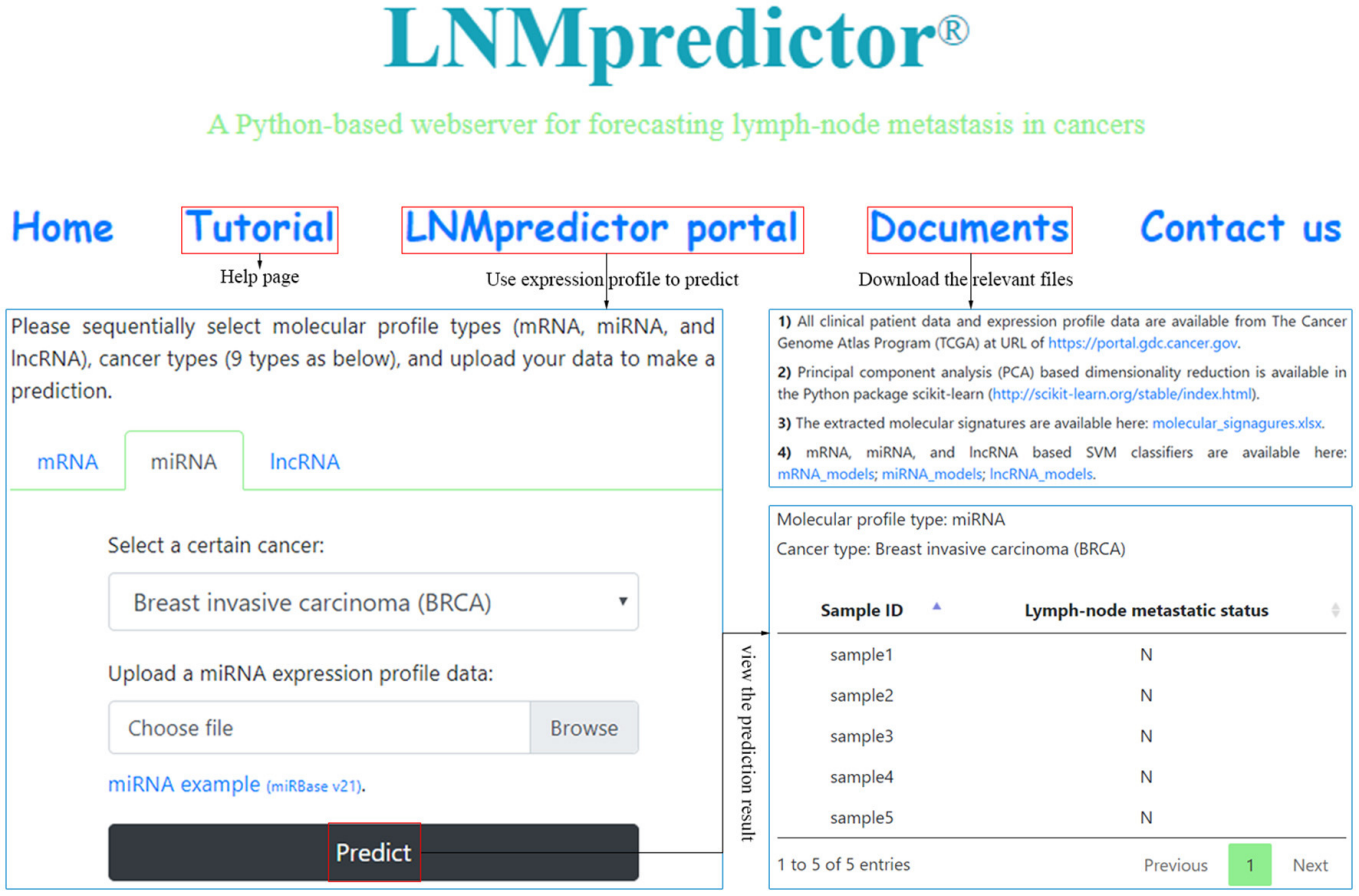
An overview of LNMpredictor that shows the tutorial, webserver portal, and document pages.

The performance of a classifier is mainly determined by the quality and the number of extracted features (Saeys et al., 2007; Tang et al., 2014). In our strategy, differentially expressed mRNAs, miRNAs, and lncRNAs in lymph-node metastasis of cancers were selected as discriminatory features for different classifiers. For miRNA-based datasets, the optimal number of differentially expressed miRNAs was gained as biomarkers from the first round of feature extraction analysis (with only biological consideration), whereas the best performance of mRNA- and lncRNA-based classifiers were gained using all the three rounds of feature extraction analysis (with both biological consideration and PCA-based feature reduction). With the optimal selection of differentially expressed biomarkers, we used the SVM algorithm to train the classifiers and generated individual models. Here, samples of lymph-node metastatic and non-metastatic groups in each cancer for different profiles were balanced and then fed to SVM classification algorithm, and all the training for different classifiers went through a fivefold cross-validation. For a comprehensive evaluation of the algorithm, we compared the performance of SVM classifiers with other two benchmark classifiers (KNN and RF). The reported prediction results of the three kinds of classifiers based on different profiles are available in Supplementary Table 2, where detailed training and testing accuracies were provided for useful information on cancer lymph-node metastasis prediction. Our results showed that the SVM classifiers slightly outperformed both the KNN and RF classifiers with an average 2% increase. Table 2 shows the fivefold cross-validation training and testing accuracy of our SVM classifiers based on different profiles for the nine cancers. The lymph-node metastatic states in cancers correctly predicted by mRNA-, miRNA-, and lncRNA-based SVM predictors accounted for the majority of all TCGA cases, with overall testing accuracies of 81.00% (mRNA-based, interval: 67.96–92.19%), 81.97% (mRNA-based, interval: 70.83–95.24%), and 80.78% (lncRNA-based, interval: 69.61–90.00%) by average small size of features (6, 12, and 4). We also made these SVM classifiers publicly available in a webserver named LNMpredictor (http://lnmpredictor.wchoda.com) that aids researchers in predicting lymph-node metastasis by uploading their expression profiles of different types (Figure 3).

## Discussion

Uncertain lymph-node metastasis in cancer diagnosis is a major limiting factor for patient survival and prognosis. A clear prediction of regional metastasis will aid in targeted tumor treatment and optimal clinical management. With massive amounts of expression profile data of different types available, machine learning methods have been widely applied in cancer prediction such as tumor origin, cancerous or non-cancerous state, and cancer subtype (Blaveri et al., 2005; Tang et al., 2018). Although attempts have focused on several cancer types using small samples of patient retrospective survey, the lymphatic metastasis evaluation of most cancers based on different profiles remained to be systematically explored with different prevalent classification algorithms, which should be conducted to improve the clinical evaluation and treatment of patients as efficient genomics diagnostic algorithms.

In this study, we applied an integrated analysis of clinical patient data (textual) and expression profile data (digital) of cancer cases. Using TNM-based staging and sample annotation information, we differentiated normal, lymph-node metastatic, and non-metastatic cases for each of the selected cancer types. Based on this, a novel feature selection strategy was proposed to identify differentially expressed mRNAs, miRNAs, and lncRNAs as discriminatory biomarkers in cancer lymph-node metastasis prediction. This feature extraction demonstrated its economy with small feature size, and also efficiency with high classification performance when used in SVM classifiers. For representative cancers, we showed that these classifiers had comparative results based on the same profiles. We also compared our SVM classifiers with other two benchmark classifiers (KNN and RF), and the results showed that the SVM classifiers had better performance. Given this, we developed a webserver that deployed SVM predictors to aid users in lymph-node metastasis forecasting by uploading their mRNA, miRNA, or lncRNA expression profiles of interested cancers.

The datasets for lymphatic metastasis prediction represented one main data regime (*v* > *s*), where *v* and *s* denote variable (i.e., gene) number and sample size, respectively. For mRNA, miRNA, and lncRNA-based dataset, *v* is much larger than *s*, which is particularly for mRNA and lncRNA cases (~2 orders of magnitude). As to miRNA profiles, the first round of feature extraction with only biological consideration ensured appropriate number of features to shape accurate classifiers. Instead, all three rounds of feature extraction, which considered both biological and mathematical aspects, was necessary for mRNA and lncRNA profiles in constructing competing classifiers. Thus, the differentiated feature extraction pipelines of different profiles depended much on nature of the data. In addition, we showed that classifiers with different profiles as well as different classification algorithms had the comparative prediction results, indicating the plasticity and efficiency of our feature extraction in lymph-node metastasis risk evaluation.

Apart from mRNA, miRNA, and lncRNA profiles, we can also consider other types of profiles (e.g., DNA methylation and protein) for lymph-node metastasis prediction. As an important epigenetic regulatory mechanism, DNA methylation has been large-scale profiled and extensively applied in cancer prediction, such as tumor classification and prognosis (Hu et al., 2019; Yang et al., 2019b). Therefore, the schedule of establishing classifiers with all profiles separated or integrated will enhance this related research. We also noted that the extracted biomarkers had strong heterogeneous property in different types of cancers, which may have specific contributions in certain lymph-node metastatic events and should be explored in further experimental studies. Moreover, international cancer sequencing projects have been performed, and the generated abundant expression profile data are accessible in ICGC [International Cancer Genome Consortium (Romeo-Casabona et al., 2012)], which may provide useful clues for improvement of prediction strategy from a cross-population perspective.

## Data Availability Statement

The original contributions presented in the study are included in the article/Supplementary Material, further inquiries can be directed to the corresponding authors.

## Author Contributions

SZ and CZ did the data analysis, developed the webserver, and wrote the paper. JD, RZ, and SY did the data analysis and anticipated the writing. BL gave useful suggestions. PW and WD supervised the project and revised the manuscript. All authors contributed to the article and approved the submitted version.

## Conflict of Interest

The authors declare that the research was conducted in the absence of any commercial or financial relationships that could be construed as a potential conflict of interest.
